# Supplementary material for: Inter-individual and inter-regional variability of breast milk antibody reactivity to bacterial lipopolysaccharides
Source: Front Immunol. 2024 Sep 6;15:1404192. doi: 10.3389/fimmu.2024.1404192 (PMC11412857; doi:10.3389/fimmu.2024.1404192)
Supplement: Supplementary file 1 [file DataSheet1.pdf]

Supplementary Material

Inter-individual and inter-regional variability of breast milk antibody reactivity to bacterial lipopolysaccharides

Lisa Crone[1], Jens Sobek[2], Nicole Müller[1], Tanja Restin[1,3], Dirk Bassler[3], Daniela Paganini[4], Michael B. Zimmermann[5], Patricia Zarnovican[6], Françoise H. Routier[6], Tais Romero-Uruñuela[7], Luis Izquierdo[7,8], Thierry Hennet[1,*]

*** Correspondence:** Thierry Hennet: Thierry.hennet@uzh.ch

1 Supplementary Figures and Tables

1.1 Supplementary Figures

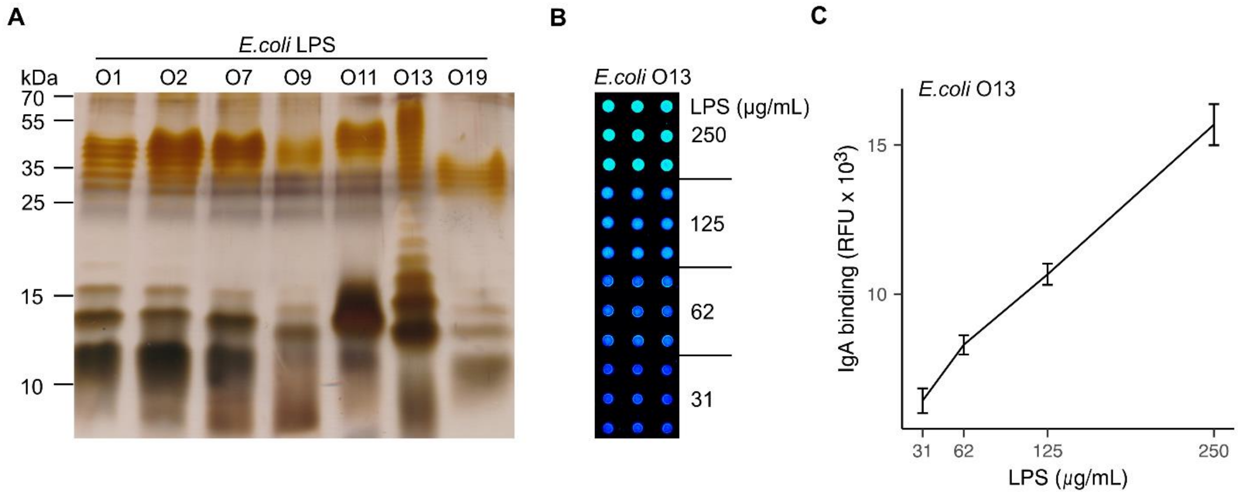

Supplementary Figure 1. LPS isolation and array printing. (A) SDS-PAGE and silver staining of LPS isolated from *E. coli serotypes*. (B) Fluorescence signal of *E. coli* O13 LPS printed in 9-dot matrices at 31, 62, 125 and 250 µg/mL visualized by scanning at 543 nm after binding to breast milk IgA and Cy3-labelled anti-human IgA antibody. (C) Quantitation of IgA binding to *E. coli* O13 LPS as mean ± S.D. of net fluorescence intensity from nine replicates per LPS concentration.

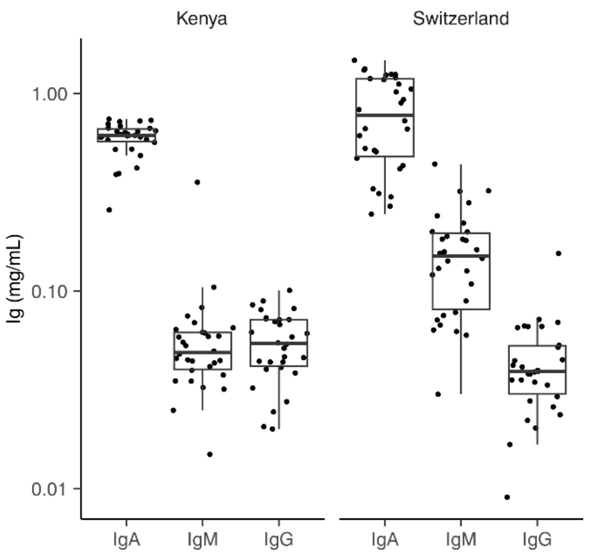

Supplementary Figure 2. Immunoglobulin concentrations in breast milk samples. IgA, IgM and IgG levels (mg/mL) in breast milk samples from Kenya and Switzerland were determined by ELISA. Unpaired Wilcoxon test used. IgM concentrations in samples from Switzerland were significantly higher than in Kenyan samples (p-value = $5.2x\ 10^{9-}$).

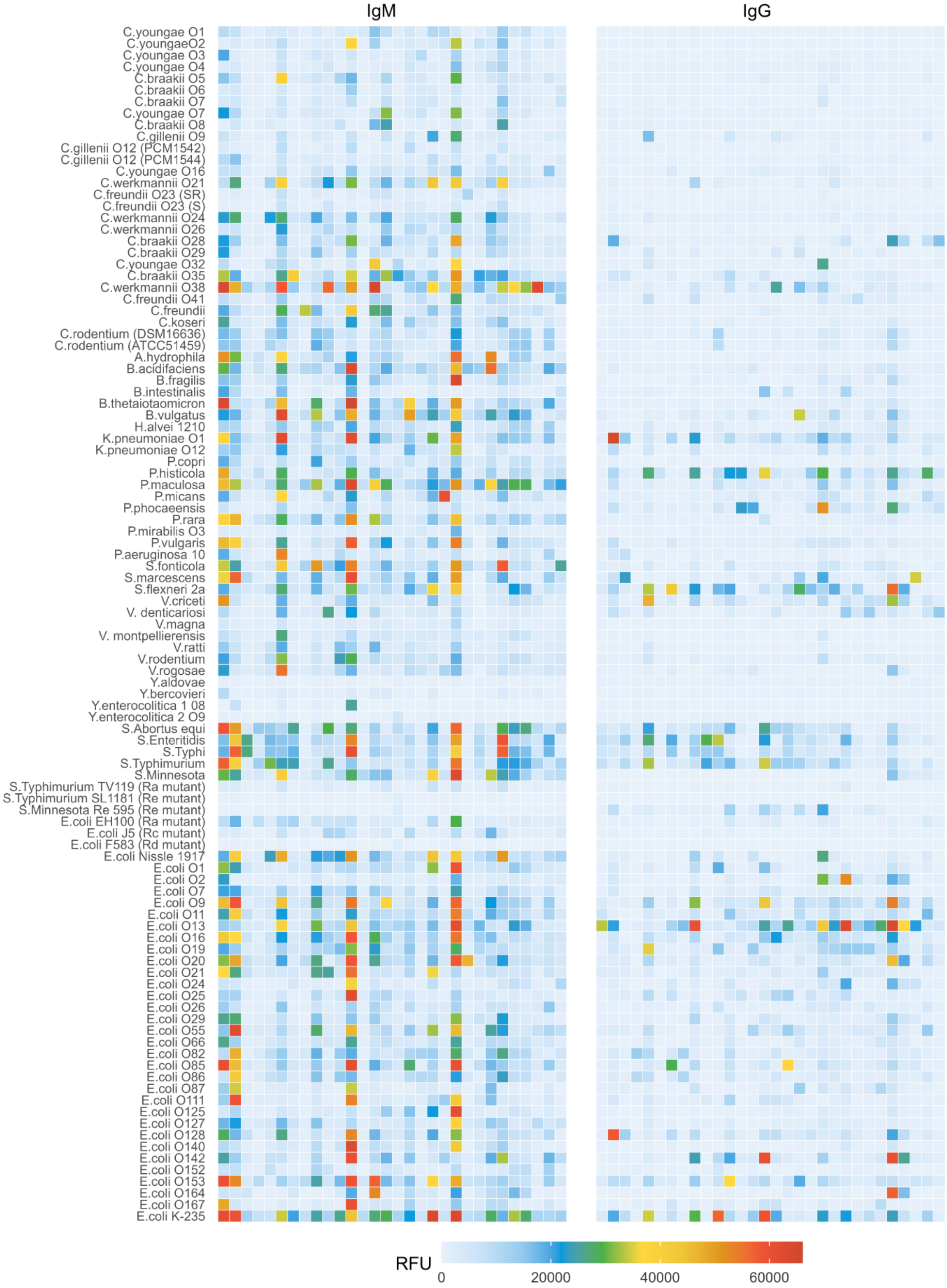

Supplementary Figure 3. Breast milk IgM and IgG binding to LPS in Kenyan samples. Heatmap of IgM reactivity (left panel) detected using anti-human IgM Cy3 and scanned at 230 V PMT-gain. Heatmap of IgG reactivity (right panel) scanned at 250 V PMT-gain. Data are shown as net mean RFU values of 9-replicate dots.

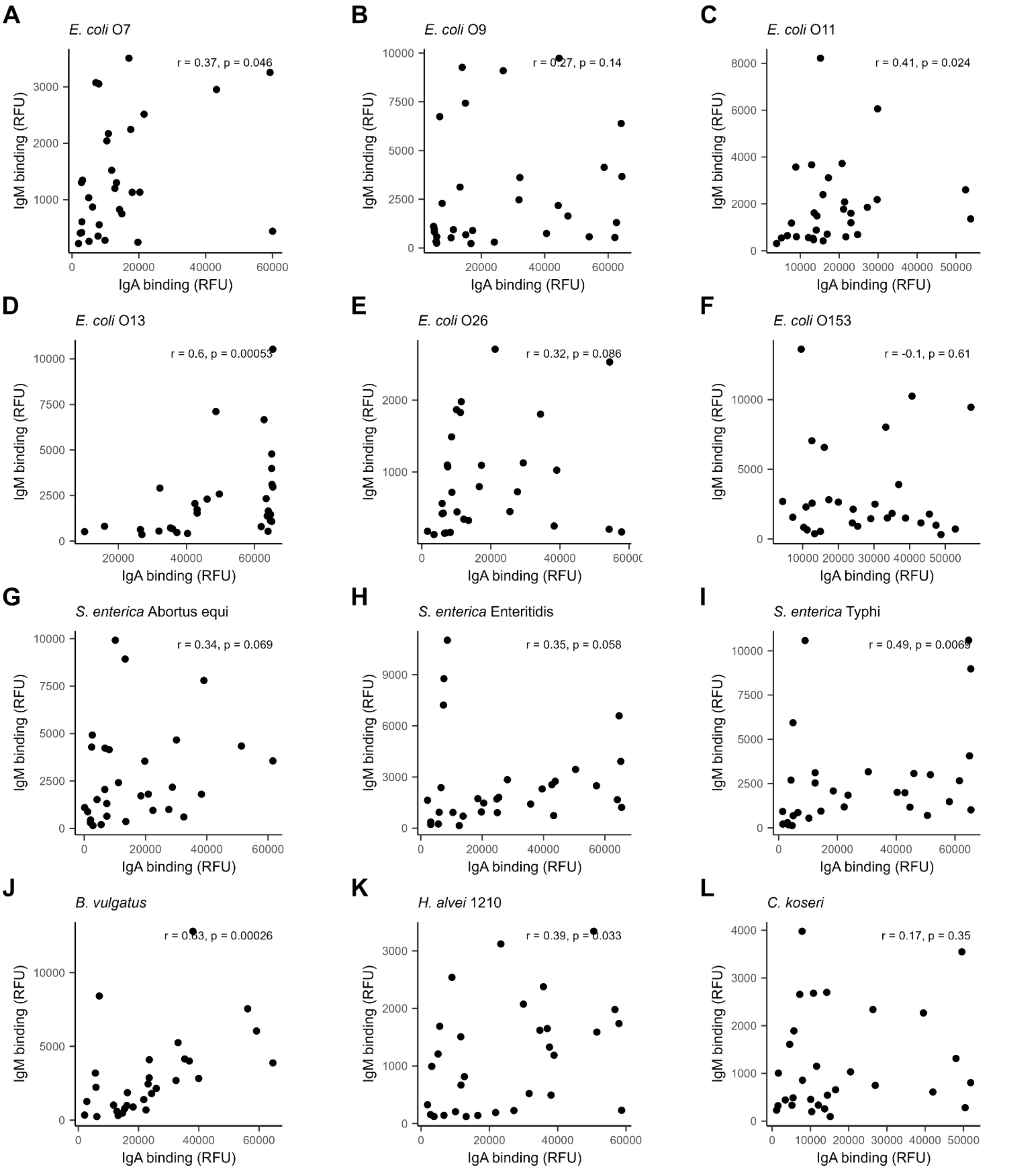

Supplementary Figure 4. Correlation of IgA and IgM binding to LPS. Data points represent individual breast milk samples from Kenya. IgA and IgM binding signals to specific LPS are given as net mean RFU. (A) *E. coli* O7, (B) *E. coli* O9, (C) *E. coli* O11, (D) *E. coli* O13, (E) *E. coli* O26, (F)*E. coli* O153, (G) *S. enterica* Abortus equi, (H) *S. enterica* Enteriditis, (I) *S. enterica* Typhi, (J) *B.*

vulgatus, (K) *H. alvei* 1210, (L) *C. koseri* LPS. Spearman correlation coefficients and p-values are listed for each panel.

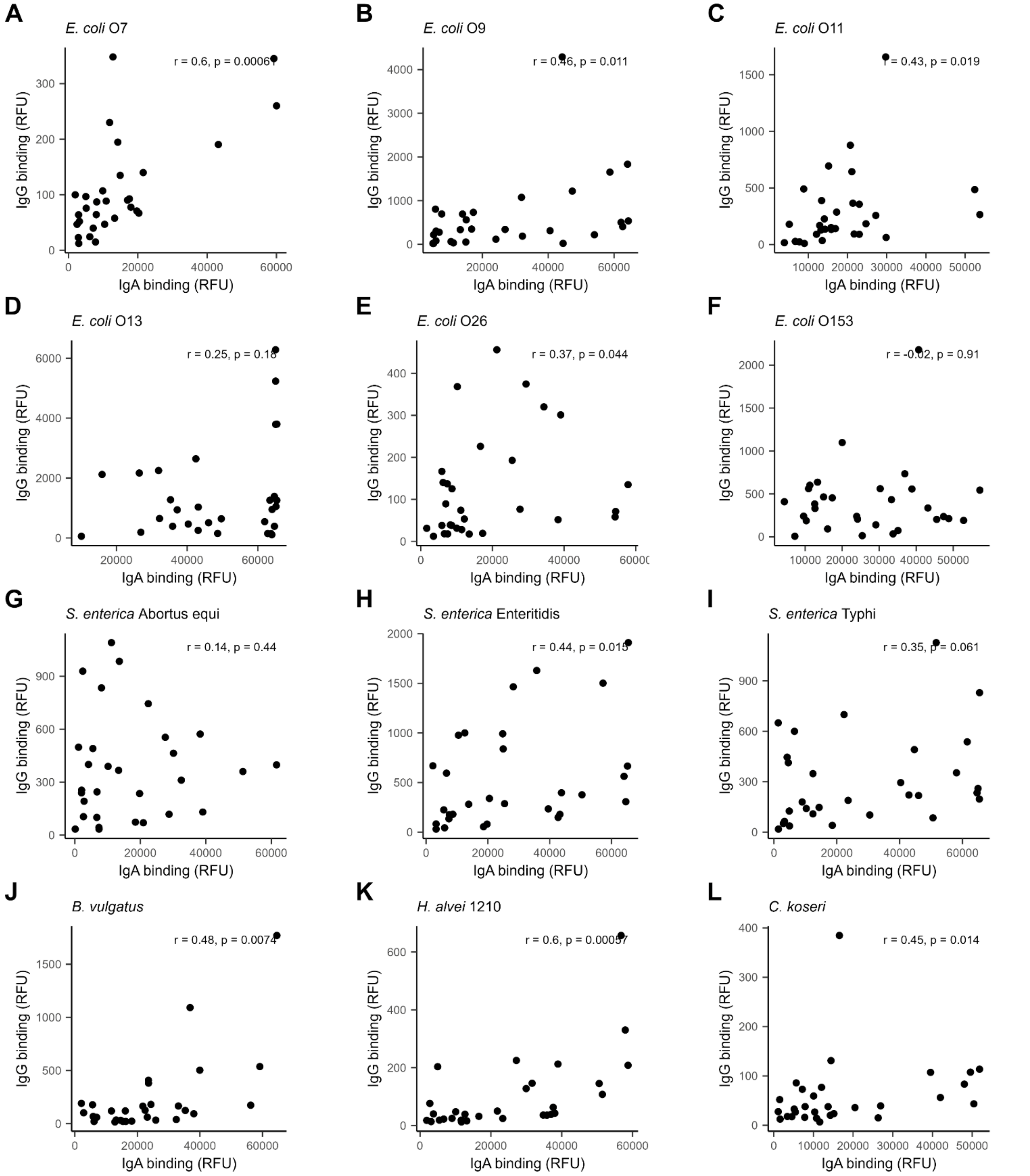

Supplementary Figure 5. Correlation of IgA and IgG binding to LPS. Data points represent individual breast milk samples from Kenya. IgA and IgG binding signals to specific LPS are given as net mean RFU. (A) *E. coli* O7, (B) *E. coli* O9, (C) *E. coli* O11, (D) *E. coli* O13, (E) *E. coli* O26, (F) *E. coli* O153, (G) *S. enterica* Abortus equi, (H) *S. enterica* Enteriditis, (I) *S. enterica* Typhi, (J) *B.*

vulgatus, (K) *H. alvei* 1210, (L) *C. koseri* LPS. Spearman correlation coefficients and p-values are listed for each panel.

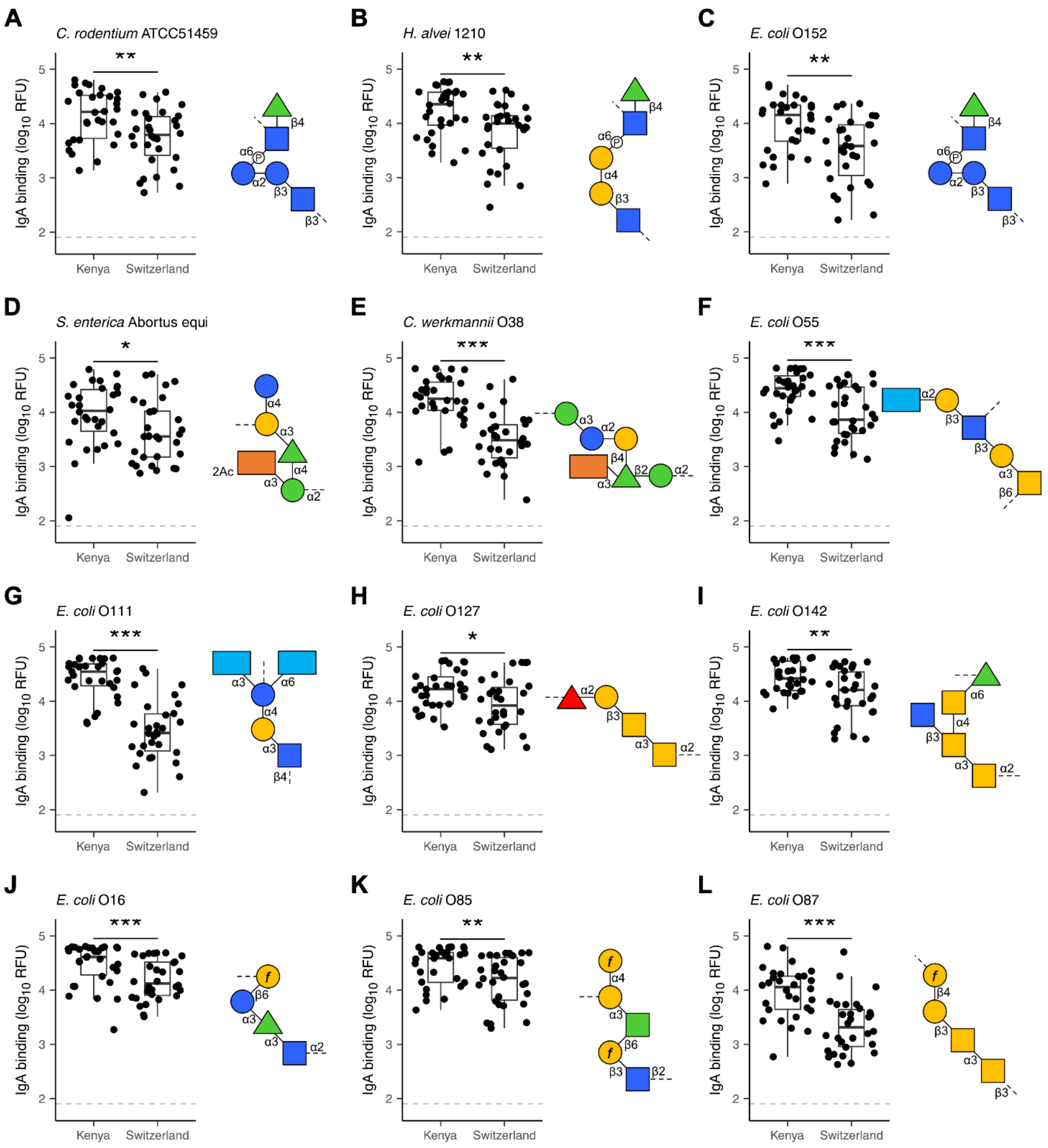

Supplementary Figure 6. Comparison of breast milk IgA reactivity towards specific LPS. LPS containing rhamnose (green triangle) as in (A) *C. rodentium*, (B) *H. alvei* 1210 and (C) *E. coli* O152 . LPS containing the 1,3-dideoxy sugars abequose (orange rectangle) and colitose (turquoise rectangle) as found in (D) *S. enterica* Abortus equi, (E) *C. werkmannii* O38, (F) *E. coli* O55 and (G) *E. coli* O111. LPS featuring the Forssman antigen (two yellow squares) as found in (H) *E. coli* O127, (I) *E. coli* O142, (L) *E. coli* O87. LPS containing galactofuranose (*f* in yellow circle) as in (J) *E. coli* O16, (K) *E. coli* O85 and (L) *E. coli* O87. IgA binding was detected using anti-human IgA Cy3, scanned at PMT-gain 190V. Comparisons of net mean RFU values between Kenyan (n=30) and Swiss (n=30)

samples using unpaired Wilcoxon test. P-values ≤ 0.05 were considered significant ("***"=0.001, "**"=0.01, "*"=0.05). The grey dotted line marks the threshold of detection (TOD).

1.2 Supplementary Tables

Supplementary Table 1. LPS library printed on arrays. Pure bacterial cultures were grown under recommended conditions (medium, temperature and oxygen requirements) by the supplier. No growth media are indicated for commercially supplied LPS. Known O-antigen structures are referenced.

**O-antigen**	**Source**	**Culture conditions**			**Structure**
		Growth medium	Temperature	Oxygen	
Aeromonas hydrophila	DSM 30187	Trypticase Soy Yeast Extract Medium (DSMZ 92)	30°C	aerobic	N/A
Bacteroides acidifaciens	DSM 100502	Chopped Meat Medium (DSMZ 78)	37°C	anaerobic	N/A
Bacteroides fragilis	DSM 2151	Chopped Meat Medium (DSMZ 78)	37°C	anaerobic	N/A
Bacteroides intestinalis	DSM 17393	modified PYG medium (DSMZ 104)	37°C	anaerobe	N/A
Bacteroides thetaiotaomicron	DSM 2079	Chopped Meat Medium (DSMZ 78) + haemin and vitamin K1	37°C	anaerobe	N/A
Bacteroides vulgatus	DSM 1447	Chopped Meat Medium (DSMZ 78) + haemin and vitamin K1	37°C	anaerobe	N/A
***Citrobacter youngae* O1**	PCM 1506	Nutrient broth (DSMZ 1)	37°C	aerobe	[1]
***Citrobacter youngae* O2**	PCM 1507	Nutrient broth (DSMZ 1)	37°C	aerobe	[1]
***Citrobacter youngae* O3**	PCM 1509	Nutrient broth (DSMZ 1)	37°C	aerobe	[1]
***Citrobacter youngae* O4**	PCM 1525	Nutrient broth (DSMZ 1)	37°C	aerobe	[1]
***Citrobacter braakii* O5**	PCM 1528	Nutrient broth (DSMZ 1)	37°C	aerobe	[1]

***Citrobacter braakii* O6**	PCM 1531	Nutrient broth (DSMZ 1)	37°C	aerobe	[1]
***Citrobacter braakii* O7**	PCM 1532	Nutrient broth (DSMZ 1)	37°C	aerobe	[1]
***Citrobacter youngae* O7**	PCM 1503	Nutrient broth (DSMZ 1)	37°C	aerobe	[1]
***Citrobacter braakii* O8**	PCM 1536	Nutrient broth (DSMZ 1)	37°C	aerobe	[1]
***Citrobacter gillenii* O9**	PCM 1537	Nutrient broth (DSMZ 1)	37°C	aerobe	[1]
***Citrobacter gillenii* O12**	PCM 1544	Nutrient broth (DSMZ 1)	37°C	aerobe	[1]
***Citrobacter gillenii* O12**	PCM 1542	Nutrient broth (DSMZ 1)	37°C	aerobe	[1]
***Citrobacter youngae* O16**	PCM 1550	Nutrient broth (DSMZ 1)	37°C	aerobe	[1]
***Citrobacter werkmanii* O21**	PCM 1554	Nutrient broth (DSMZ 1)	37°C	aerobe	[1]
***Citrobacter freundii* O23 (SR)**	PCM 1556	Nutrient broth (DSMZ 1)	37°C	aerobe	[1]
***Citrobacter freundii* O23 (S)**	PCM 2352	Nutrient broth (DSMZ 1)	37°C	aerobe	[1]
***Citrobacter werkmanii* O24**	PCM 1557	Nutrient broth (DSMZ 1)	37°C	aerobe	[1]
***Citrobacter werkmanii* O26**	PCM 1559	Nutrient broth (DSMZ 1)	37°C	aerobe	[1]
***Citrobacter braakii* O28**	PCM 1561	Nutrient broth (DSMZ 1)	37°C	aerobe	[1]
***Citrobacter braakii* O29**	PCM 1562	Nutrient broth (DSMZ 1)	37°C	aerobe	[1]
***Citrobacter youngae* O32**	PCM 1569	Nutrient broth (DSMZ 1)	37°C	aerobe	[1]

***Citrobacter braakii* O35**	PCM 1586	Nutrient broth (DSMZ 1)	37°C	aerobe	[1]
***Citrobacter werkmanii* O38**	PCM 1489	Nutrient broth (DSMZ 1)	37°C	aerobe	[1]
***Citrobacter freundii* O41**	PCM 1444	Nutrient broth (DSMZ 1)	37°C	aerobe	[1]
Citrobacter freundii	DSM 30039	Trypticase Soy Yeast Extract Medium (DSMZ 92)	30°C	aerobe	N/A
Citrobacter koseri	DSM 4595	Nutrient broth (DSMZ 1)	30°C	aeroba	N/A
Citrobacter rodentium	DSM 16636	Nutrient broth (DSMZ 1)	37°C	aeroba	N/A
Citrobacter rodentium	ATCC 51459	LB broth (DSMZ 381)	37°C	aerobe	[1]
***Escherichia coli* EH100**	L9641	-	-	-	Ra rough mutant
***Escherichia coli* J5**	L5014	-	-	-	Rc rough mutant
***Escherichia coli* F583**	L6893	-	-	-	Rd rough mutant
***Escherichia coli* K-235**	L2143	-	-	-	N/A
***Escherichia coli* Nissle 1917**		LB broth (DSMZ 381)	37°C	aerobe	[2]
***Escherichia coli* O1**	DSM 10728	LB broth (DSMZ 381)	37°C	aerobe	[3]
***Escherichia coli* O2**	DSM 10772	LB broth (DSMZ 381)	37°C	aerobe	[3]
***Escherichia coli* O7**	DSM 10782	LB broth (DSMZ 381)	37°C	aerobe	[3]
***Escherichia coli* O9**	NCTC 9009	LB broth (DSMZ 381)	37°C	aerobe	[3]

***Escherichia coli* O11**	DSM 11751	LB broth (DSMZ 381)	37°C	aerobe	[3]
***Escherichia coli* O13**	DSM 1328	LB broth (DSMZ 381)	37°C	aerobe	[3]
***Escherichia coli* 016**	NCTC9016	LB broth (DSMZ 381)	37°C	aerobe	[3]
***Escherichia coli* O19**	DSM 11752	LB broth (DSMZ 381)	37°C	aerobe	[3]
***Escherichia coli* O20**	NCTC 9020	LB broth (DSMZ 381)	37°C	aerobe	[3]
***Escherichia coli* O21**	NCTC9021	LB broth (DSMZ 381)	37°C	aerobe	[3]
***Escherichia coli* O24**	NCTC 9024	LB broth (DSMZ 381)	37°C	aerobe	[3]
***Escherichia coli* O25**	DSM 22664	LB broth (DSMZ 381)	37°C	aerobe	[3]
***Escherichia coli* O26**	L8274	-	-	-	[3]
***Escherichia coli* O29**	DSM 9026	LB broth (DSMZ 381)	37°C	aerobe	[3]
***Escherichia coli* O55**	L2880	-	-	-	[3]
***Escherichia coli* O66**	NCTC 9066	LB broth (DSMZ 381)	37°C	aerobe	[3]
***Escherichia coli* O82**	NCTC 9082	LB broth (DSMZ 381)	37°C	aerobe	[3]
***Escherichia coli* O85**	NCTC 9085	LB broth (DSMZ 381)	37°C	aerobe	[3]
***Escherichia coli* O86**	NCTC 9086	LB broth (DSMZ 381)	37°C	aerobe	[3]
***Escherichia coli* O87**	NCTC 9087	LB broth (DSMZ 381)	37°C	aerobe	[3]

***Escherichia coli* O111**	L2630	-	-	-	[3]
***Escherichia coli* O125**	DSM 8700	LB broth (DSMZ 381)	37°C	aerobe	[3]
***Escherichia coli* O127**	L3129	-	-	-	[3]
***Escherichia coli* O128**	L2755	-	-	-	[3]
***Escherichia coli* O140**	NCTC 10087	LB broth (DSMZ 381)	37°C	aerobe	[3]
***Escherichia coli* O142**	DSM 9024	LB broth (DSMZ 381)	37°C	aerobe	[3]
***Escherichia coli* O152**	DSM 9030	LB broth (DSMZ 381)	37°C	aerobe	[3]
***Escherichia coli* O153**	NCTC 10960	LB broth (DSMZ 381)	37°C	aerobe	[3]
***Escherichia coli* O164**	NCTC 10361	LB broth (DSMZ 381)	37°C	aerobe	[3]
***Escherichia coli* O167**	DSM 9033	LB broth (DSMZ 381)	37°C	aerobe	[3]
***Hafnia alvei* 1210**	PCM 1210	Nutrient broth (DSMZ 1)	37°C	aerobe	[4]
***Klebsiella pneumoniae* O1**	L4268	-	-	-	[5]
***Klebsiella pneumoniae* O12**	PCM 80	Nutrient broth (DSMZ 1)	37°C	aerobe	[5]
Prevotella copri	DSM 18205	Schaedler broth (Roth;5772)	37°C	anaerobe	N/A
Prevotella histicola	DSM 19854	modified PYG medium (DSMZ 104)	37°C	anaerobe	N/A
Prevotella maculosa	DSM 19339	modified PYG medium (DSMZ 104)	37°C	anaerobe	N/A

Prevotella micans	DSM 21469	modified PYG medium (DSMZ 104) + 5 % horse serum	37°C	anaerobe	N/A
Prevotella phocaeensis	DSM 103364	Chopped meat medium (DSMZ 78) + haemin and vitamin K1	37°C	anaerobe	N/A
Prevotella rara	DSM 105141	Chopped meat medium (DSMZ 78) + haemin and vitamin K1	37°C	anaerobe	N/A
Prevotella salivae	DSM 15606	Chopped meat medium (DSMZ 78)	37°C	anaerobe	N/A
Proteus vulgaris	SMB00801	-	-	-	N/A
***Pseudomonas aeruginosa* 10**	L9143	-	-	-	N/A
***Salmonella enterica* Abortus equi**	L5886	-	-	-	[6]
***Salmonella enterica* Enteritidis**	L6011	-	-	-	[6]
***Salmonella enterica* Minnesota**	L6261	-	-	-	N/A
***Salmonella entérica* Minnesota Re 595**	L9764	-	-	-	Re rough mutant
***Salmonella enterica* Typhimurium**	L6511	-	-	-	[6]
***Salmonella enterica* Typhimurium SL1181**	L9516	-	-	-	Re rough mutant
***Salmonella enterica* Typhimurium TV119**	L6016	-	-	-	Ra rough mutant

***Salmonella enterica* Typhi**	L6386	-	-	-	[6]
Serratia fonticola	DSM 103300	Nutrient broth (DSMZ 1)	28°C	aerobe	N/A
Serratia marcescens	L6136	-	-	-	N/A
***Shigella flexneri* 2a**	DSM 4782	Trypticase Soy Yeast Extract Medium (DSMZ 92)	37°C	aerobe	[7]
Veillonella criceti	DSM 20734	Veillonella medium (DSMZ 136)	37°C	anaerobe	N/A
Veillonella denticariosi	DSM 19009	Veillonella medium (DSMZ 136)	37°C	anaerobe	N/A
Veillonella magna	DSM 19857	modified PYG medium (DSMZ 104) + 0.5 - 1% lactate	37°C	anaerobe	N/A
Veillonella montpellierensis	DSM 17217	modified PYG medium (DSMZ 104) + 10 µl/mL putrescine (0.03 %) + 10 µl/mL sodium lactate (50 %)	37°C	anaerobe	N/A
Veillonella ratti	DSM 20736	Veillonella medium (DSMZ 136)	37°C	anaerobe	N/A
Veillonella rodentium	DSM 20737	Veillonella medium (DSMZ 136)	37°C	anaerobe	N/A
Veillonella rogosae	DSM 18960	modified PYG medium (DSMZ 104) + 0.5 – 1 % lactate	37°C	anaerobe	N/A
Yersinia aldovae	DSM 18303	Trypticase Soy broth (Oxoid CM0129)	30°C	aerobe	N/A
Yersinia bercovieri	DSM 18528	Trypticase Soy broth (Oxoid CM0129)	30°C	aerobe	N/A
***Yersinia enterocolitica* O8**	DSM 27689	BHI medium (Sigma-Aldrich; 53286)	28°C	aerobe	[8]

***Yersinia enterocolitica* O9**	DSM 9499	BHI medium (Sigma-Aldrich; 53286)	28°C	aerobe	[8]

1. Knirel, Y.A., et al., *Structures and serology of the O-specific polysaccharides of bacteria of the genus Citrobacter.* Archivum immunologiae et therapiae experimentalis, 2002. **50**(6): p. 379–391.
2. Blum, G., R. Marre, and J. Hacker, *Properties of Escherichia coli strains of serotype O6.* Infection, 1995. **23**(4): p. 234–236.
3. Stenutz, R., A. Weintraub, and G. Widmalm, *The structures of Escherichia coli O-polysaccharide antigens.* FEMS microbiology reviews, 2006. **30**(3): p. 382–403.
4. Romanowska, E., *Immunochemical aspects of Hafnia alvei O antigens.* FEMS immunology and medical microbiology, 2000. **27**(3): p. 219–225.
5. Vinogradov, E., et al., *Structures of lipopolysaccharides from Klebsiella pneumoniae. Eluicidation of the structure of the linkage region between core and polysaccharide O chain and identification of the residues at the non-reducing termini of the O chains.* Journal of Biological Chemistry, 2002. **277**(28): p. 25070–25081.
6. Liu, B., et al., *Structural diversity in Salmonella O antigens and its genetic basis.* FEMS microbiology reviews, 2014. **38**(1): p. 56–89.
7. Perepelov, A.V., et al., *Shigella flexneri O-antigens revisited: final elucidation of the O-acetylation profiles and a survey of the O-antigen structure diversity.* FEMS Immunology & Medical Microbiology, 2012. **66**(2): p. 201–210.
8. Bruneteau, M. and S. Minka, *Lipopolysaccharides of bacterial pathogens from the genus Yersinia: a mini-review.* Biochimie, 2003. **85**(1-2): p. 145–152.

Supplementary Table 2. LPS Overview of breast milk samples. Breast milk samples used in this stud originated from Switzerland or Kenya, maternal age range and the IgA, IgM and IgG concentrations (mg/mL) determined with ELISA.

		Antibody concentrations (mg/mL)		
Origin	Maternal age (years)	IgA	IgM	IgG
Kenya	18-40	0.25 - 0.74	0.01 - 0.35	0.02 - 0.10
Switzerland	23-41	0.24 - 1.47	0.03 - 0.44	0.01 - 0.15
